# *Culicoides insignis* Lutz, 1913 (Diptera: Ceratopogonidae) Biting Midges in Northeast of Brazil

**DOI:** 10.3390/insects12040366

**Published:** 2021-04-20

**Authors:** Raisa Rodrigues Santos Rios, Maria Clara Alves Santarém, Karlos Antônio Lisboa Ribeiro Júnior, Breno Araujo de Melo, Sybelle Georgia Mesquita da Silva, Neuriane Cavalcante da Silva, Vitória Regina Viana dos Santos, Jakeline Maria dos Santos, Antônio Euzébio Goulart Santana, Angelina Bossi Fraga

**Affiliations:** 1 Northeast Network of Biotechnology—RENORBIO, Federal University of Alagoas, Av. Lourival Melo Mota S/N, Tabuleiro dos Martins, 57072-900 Maceió, Brazil; raisa.rios@hotmail.com (R.R.S.R.); breno_melo13@hotmail.com (B.A.d.M.); belle_mesquita21@hotmail.com (S.G.M.d.S.); 2 Ceratopogonidae Collection, Oswaldo Cruz Foundation (FIOCRUZ), Av. Brasil, Manguinhos, 4365 Rio de Janeiro, Brazil; mariaclarasantarem@gmail.com; 3 Campus of Engineering and Agrarian Science—CECA, Federal University of Alagoas, BR-104, 57100-000 Rio Largo, Brazil; karloslisboa@gmail.com (K.A.L.R.J.); neurianecavalcante@gmail.com (N.C.d.S.); vihfirmino@gmail.com (V.R.V.d.S.); jackbilu@hotmail.com (J.M.d.S.); aegsal@gmail.com (A.E.G.S.)

**Keywords:** *Culicoides*, hematophagous insects, light traps

## Abstract

**Simple Summary:**

*Culicoides* genus insects are spread throughout the world, and some species are vectors of important human and animal diseases. Genetic identification, as well as the local occurrence of these insects, are fundamental to the development of risk profiles and entomological surveillance of transmitted diseases. We investigated the *Culicoides* occurrence in Alagoas State, northeastern Brazil. Midges were captured with light traps, being morphologic and genetic identified. After PCR analysis and GenBank database comparison, it was confirmed that the captured midges belong to *Culicoides insignis*. This was the first formal report of *Culicoides* insignis occurrence in Alagoas State, northeastern Brazil.

**Abstract:**

The species of the *Culicoides* genus are hematophagous, and some of them are vectors of important human and animal diseases. This group of insects is distributed worldwide, varying according to local species. Knowledge of the geographic distribution of specific species is crucial for the development and implementation of control strategies. The aim of this work was to investigate the occurrence of *Culicoides* in the state of Alagoas in northeast Brazil. Midges were captured with CDC light traps, and their identification and morphological analyses were performed by the Ceratopogonidae Collection of the Oswaldo Cruz Foundation (FIOCRUZ/CCER) in Rio de Janeiro, Brazil. Morphological analyses were performed using the key to *Culicoides* from the guttatus group and comparison with other deposited specimens. DNA sequencing, genetic analysis and comparison with sequences in the Genbank database, confirmed the identification of the flies as *Culicoides insignis*. This was the first formal report of *C. insignis* being found in Alagoas.

## 1. Introduction

Members of the genus *Culicoides* Latreille, 1809 are Diptera of the family Ceratopogonidae. Also known as biting midges or no-see-ums, these are among the most important and the smallest members of this family, measuring approximately 1.5 to 5 mm in length. Their wings have a pattern of dark and pale spots, which aid in species identification. They are one of the most important hematophagous insects in this family and females of some *Culicoides* species can transmit, through their bites, the causative agents of important veterinary diseases, including Bluetongue [1] Schmallenberg [2] African horse sickness [3] epizootic hemorrhagic disease (EHD), equine encephalosis (EE), Akabane (AKA), and bovine ephemeral fever (BEF) [4]. The main symptoms of these diseases include edema, fever, and ulceration [5]. Additionally, *Culicoides paraensis* (Goeldi) is recognized as the vector of the Oropouche virus, which infects humans primarily in the Brazilian Amazon Region [6].

These insects are widely distributed across the globe, being reported in diverse environments and up to 4000 m of altitude [4]. The impact of these insects on public health, as important disease vectors, was highlighted by the use of models to predict the presence and abundance of *C. imicola*, the most important vector of BTV, in both Europe and Africa [7]. A modeling work conducted in Argentina evaluated the occurrence and distribution of *C. insignis*, in order to develop risk profiles for BTV [8].

In Brazil, 299 species of *Culicoides* were reported, most of them in the Amazon Region. In Northeast Brazil, no Ceratopogonidae species have ever been reported in the states of Alagoas and Sergipe [9]. The identification of the species of vectors plays an important role in determining the epidemiology and pathogens transmission. Small differences in the biology and ecology of systematically closely related species may have large effects on the probability of transmission of disease. Therefore, the accurate identification of species is basic to understanding the epidemiology of disease transmission [10]. This work aimed to investigate the occurrence of the *Culicoides* in the state of Alagoas, in northeast Brazil.

## 2. Materials and Methods

### 2.1. Morphological Identification

Insects were collected between June 2019 and September 2019 in the rainy season at Satuba city, Alagoas State, northeastern Brazil (09°33′46″ E; 35°49′26″ W). Midges were captured with CDC (Center on Disease Control) light traps, powered by electricity, that were put five days per week at the sheepfolds in twilight (5 pm) and picked up in the morning (7 am) the next day. After that, the insects were preserved in 70% ethanol solution, taken to the laboratory, and rinsed in distilled water. The specimens were slide mounted as described [11] except for fixation in Hoyer solution. The slide-mounted species were photomicrographed using a NIKON Eclipse E-200 microscope. Morphological identification was performed by the Fiocruz Ceratopogonidae Collection, Rio de Janeiro, Brazil, using the key to *Culicoides* from the guttatus group [12] and comparison with other deposited specimens.

### 2.2. DNA Extraction, COI (Cytochrome Oxidase I) Gene Amplification and Gene Submission to GenBank

Total nucleic acids were extracted following a modified Chelex method. *Culicoides* spp adults were crushed and homogenized in 20 μL of 6% Chelex solution in a 0.2 mL Eppendorf tube. The tube was agitated for a few seconds and then incubated at 57 °C for 15 min and at 99 °C for 3 min. After centrifugation at 13,000 rpm for 5 min, the supernatant was collected and used as a template for PCR amplification [13].

The PCRs were performed on a T100 thermal cycler (BioRad, Foster City, CA, USA) adjusted to the following thermal conditions: DNA denaturation and polymerase activation at 94 °C for 2 min, followed by 30 cycles at 95 °C for 30 s (denaturation), 48 °C for 45 s (annealing), and 72 °C for 45 s (extension), and 72 °C for 10 min (final elongation). The PCR products were subjected to electrophoresis on a 1.0% agarose gel and stained with SYBR^®^ Gold for visualization [14]. PCR products amplified from mtCOI of *Culicoides* spp were purified using a QIAquick Gel Extraction Kit (Qiagen Inc, Valencia, CA, USA) and sequenced using a Macrogen instrument (Macrogen Inc, Seoul, South Korea) in both directions using the forward primer (LCO1490) 5′-GGTCAACAAATCATAAAGATATTGG-3′ and reverse primer (HCO2198) 5′-TAAACTTCAGGGTGACCAAAAAATCA-3′ [15].

Sequenced data were checked for quality by Codon Code Aligner v. 6.0.2 (www.codoncode.com) (Codon Code Corporation, Dedham, MA, USA). Homology, insertions—deletions, stop codons, and frameshifts were checked using NCBI BLAST. BankIt, a WWW-based submission tool with wizards to guide the submission process, was used. The GenBank database is intended for new sequence data that were determined and annotated by the submitter. All sequences were uploaded to GenBank.

### 2.3. Data Analysis

The determined sequences were manually edited with Codon Code^®^ (version 6.0.2) to produce a consensus sequence of ∼670 bp. The sequences were aligned using the multiple sequence comparison by log expectation (MUSCLE) program in Mega, version 10.1.7 [16]. The sequences were compared with publicly available sequences in the Barcode of Life Data System (BOLD) database to establish their relationship [17].

## 3. Results and Discussion

### 3.1. Morphological Identification

**Figure 1:**
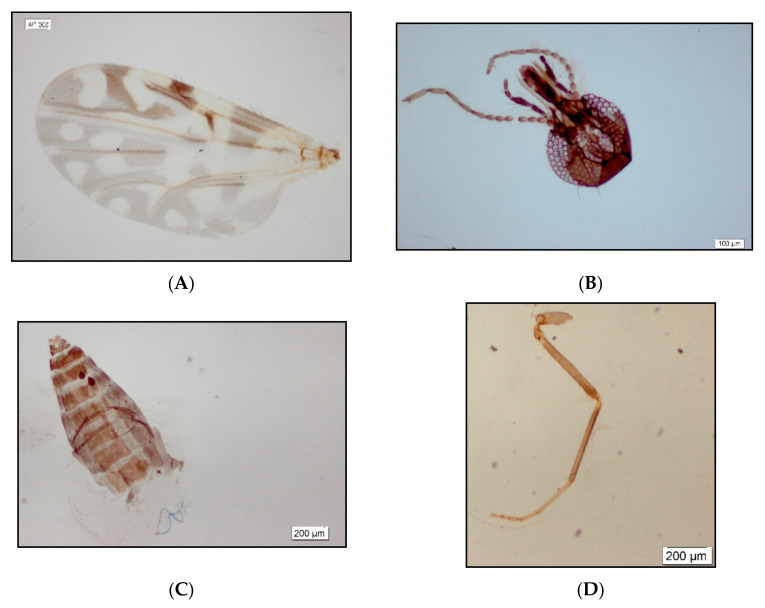
Body parts of *Culicoides insignis* Lutz, 1913: female, (**A**) wings; (**B**) head, anterior view; (**C**) abdomen, ventral view; (**D**) midleg.

A total of 741 specimens (518 males and 223 females) were collected, and all of them belonged to only one species, identified by the Fiocruz Ceratopogonidae Collection as *C. insignis* Lutz, 1913 (Figure 1). This was the first formal record of Ceratopogonidae in Alagoas, Brazil. Since the *Culicoides* genus has been reported in almost all Brazilian states, except in the states of Alagoas, Tocantins, Rio Grande Norte, Sergipe and Distrito Federal [9]. Additionally, little is known about the *Culicoides* fauna in Caatinga, an important and biodiverse Brazilian biome.

The occurrence of this species in a large number of farm environments near sheep farming is an important epidemiological record. *Culicoides insignis* is the main vector of Bluetongue virus (BTV) in the Neotropical Region to domestic and wild ruminants in the Neotropical region [18]. This species is essentially zoophilic and is often associated with cattle [19]. It is also found in manure and wet pasture areas, as well as mangroves, stream banks and sugar cane plantations [20]. This behavior highlights its importance as vectors of this disease to these animals, as well as in allergic dermatitis caused by their bite. Its distribution occurs throughout the Neotropical Region up to the south of Florida, with a peak population in rainy and hot seasons, which may constitute almost the totality of species captured in this period in areas with available hosts. *Culicoides insignis* has been shown to be a competent vector of serotypes BTV 2 and 11 in Neotropical areas [21]. This fact suggests that *C. insignis* may act as a vehicle for the expansion of serotypes from the Caribbean, Central America and south Florida and further north and for the expansion of serotypes from the northern USA to the south [22].

### 3.2. Molecular Identification

To determine the relationship of the identified species within the genus *Culicoides*, maximum likelihood analysis of the 645-bp COI sequences (after removing the 5′- and 3′-end regions) was conducted using the 30 sequences identified in this study and the most identical sequences of corresponding *Culicoides* species obtained from the BOLD database (Table 1). According to the subgeneric classification of [23] the species identified in this study were classified into the Ceratopogoninae subfamily, Culicoidini tribe and *Culicoides* genus.

The sequences obtained in the present study have been deposited in GenBank under the accession numbers *C. insignis* MT806183 and MW871560-MW871562. All the species from north America showed in Table 2 were at least 79% identical to specimens reported here. The genetic distance pairwise between *C. insignis* from Brazil and *C. sonorensis*, *C. haematopotus*, and *C. stellifer*, were 82.80%, 82.10% and 80.00%, respectively (Table 2). Highlighting, the estimations between the genetic distance pairwise from *Culicoides* midges from America, as showed in Table 2, have similar genetic homology to our finds. For example, *C. sonorensis* was 84.00% identical to *C. stellifer*. A recent paper discussed the expansion of the range of *C. insignis* in Florida and the southeastern U.S. [24]. Although *C. insignis* is more widespread in Brazil, this is considered yet another example of the expansion of its range.

This is the first sequence of the COI gene from *Culicoides insignis*, and it is the first DNA fragment sequence from an important widespread Brazilian species. These data can collaborate with studies on *Culicoides* genetic diversity in Brazil and provide support for epidemiological studies on Bluetongue virus transmission.

## 4. Conclusions

This is the first report of a member of the Ceratopogonidae, *Culicoides insignis*, in Alagoas, Brazil, and first report of the COI gene sequence for this species in Brazil.

## Figures and Tables

**Table 1:** Sequence ID of 30 COI sequences of collected *Culicoides* midges in various regions of America and their identity searched in the BOLD system database.

Species	Sequence ID	Collection Site	Country
*Culicoides sonorensis*	BBDIT1920-12	Texas	United States
*Culicoides sonorensis*	BBDIV1423-12	California	United States
*Culicoides sonorensis*	BBDIV281-12	California	United States
*Culicoides sonorensis*	BBDIV461-12	California	United States
*Culicoides sonorensis*	BBDIV647-12	New mexico	United States
*Culicoides sonorensis*	CNGRD391-12	Saskatchewan	Canada
*Culicoides sonorensis*	CNGSB2323-12	Saskatchewan	Canada
*Culicoides haematopotus*	GBMNA39319-19	Texas	United States
*Culicoides haematopotus*	GBMNA39320-19	Texas	United States
*Culicoides haematopotus*	GBMNA39321-19	Texas	United States
*Culicoides haematopotus*	GBMNA39322-19	Texas	United States
*Culicoides haematopotus*	GBMNA39323-19	Texas	United States
*Culicoides haematopotus*	GBMNA39330-19	Texas	United States
*Culicoides haematopotus*	GBMNA39332-19	Texas	United States
*Culicoides stellifer*	GBMNB14874-20	Texas	United States
*Culicoides stellifer*	GBMNB14875-20	Texas	United States
*Culicoides stellifer*	BARSD143-16	Ontario	Canada
*Culicoides stellifer*	BARSE114-16	Ontario	Canada
*Culicoides stellifer*	GBMNB14876-20	Texas	United States
*Culicoides stellifer*	GBMNB14877-20	Texas	United States
*Culicoides stellifer*	GBMNB14878-20	Texas	United States
*Culicoides stellifer*	GBMNB14879-20	Texas	United States

**Table 2:** Estimation of pairwise distances between sampled species for COI of the mtDNA.

	1	2	3	4	5	6
1. *C. insignis (MT806183)*						
2. *C. sonorensis* (*BBDIV1423)*	0.172					
3. *C. sonorensis* (*BBDIV461)*	0.174	0.004				
4. *C. haematopotus (GBMNA39330)*	0.179	0.212	0.217			
5. *C. haematopotus (GBMNA39332)*	0.182	0.209	0.213	0.012		
6. *C. stellifer (GBMNB14874)*	0.200	0.170	0.157	0.220	0.224	
7. *C. stellifer (GBMNB14876)*	0.200	0.174	0.157	0.220	0.224	0.182

## Data Availability

The data generated during the study have already been reported in the manuscript.
